# Enhancing accessibility to water treatment: An economical and sustainable method for producing a moringa oleifera-based natural coagulant through paper filtration

**DOI:** 10.1016/j.mex.2024.103060

**Published:** 2024-11-26

**Authors:** Danieli Soares de Oliveira, Raynara Souza do Nascimento, Clainer Bravin Donadel

**Affiliations:** a Federal Institute of Espírito Santo - campus Cariacica, Rodovia Governador José Sette, 184, 29150-410, Cariacica, Espírito Santo, Brazil; b Federal Institute of Espírito Santo - campus Vitória, Avenida Vitória, 1.729, 29040-780, Vitória, Espírito Santo, Brazil

**Keywords:** Cost-Effective, Rapid, and Sustainable Production of a Coagulant from Moringa oleifera Seeds for Water Treatment, Natural coagulants, Moringa oleifera, Water treatment, Turbidity removal

## Abstract

Access to safe drinking water is a major challenge for vulnerable populations, especially in regions with limited infrastructure. The use of chemical coagulants in water treatment presents environmental and health risks due to their non-biodegradable byproducts, which contaminate ecosystems. Natural coagulants offer a safer alternative, as they decompose naturally and reduce pollution. However, their production often requires complex methods, such as microfiltration and lyophilization, which are unsuitable for resource-limited areas. This study introduces a simplified method for producing a natural coagulant from the seeds of Moringa oleifera, eliminating the need for complex techniques or saline solutions. The process involves peeling, grinding the seeds, stirring with water, and filtering through paper filters, producing the coagulant in under 30 min. Coagulation tests showed complete turbidity removal with 5 mL of coagulant per liter of water at 100 NTU. This economical and environmentally sustainable method offers a practical solution for improving water treatment in resource-limited communities.

• Simplified production method for Moringa oleifera-based coagulant without complex techniques or chemical additives.
• Coagulant production is completed in <30 min, compared to hours or days in alternative methods.
• Achieves 100 % turbidity removal using only 5 mL of coagulant per liter of water in coagulation tests.

## Graphical abstract

**Figure:**
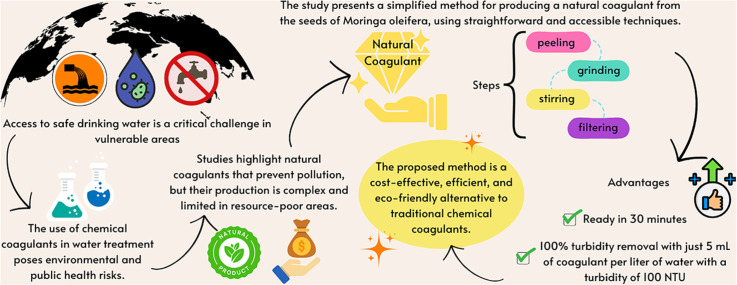

Specifications table

**Table:** 

Subject area:	Engineering
More specific subject area:	Environmental Engineering
Name of your method:	Cost-Effective, Rapid, and Sustainable Production of a Coagulant from Moringa oleifera Seeds for Water Treatment
Name and reference of original method:	Not applicable
Resource availability:	Materials: Moringa oleifera seeds, tap water, and bentonite. Equipment: mortar and pestle, magnetic stirrer, filter paper, balance, beakers, graduated pipettes, and jar test apparatus.

## Background

Access to clean water is a fundamental necessity for the health and well-being of populations globally. However, millions of people, particularly those residing in regions with limited infrastructure, continue to face significant challenges in securing clean and safe water for consumption. According to Reference [1], clean water for drinking and domestic use remains inaccessible to over 1.1 billion people worldwide.

Coagulation is a critical stage in water treatment for the removal of suspended particles, heavy metals, and other contaminants. The most widely used and conventional method involves the use of chemical-based coagulants, as highlighted by [2]. While effective, the extensive use of these chemicals raises significant environmental and health concerns, particularly due to the presence of residual metals in treated water, which are linked to health risks, including the potential onset of neurological diseases [3]. In contrast, natural coagulants represent a promising and sustainable alternative, offering efficacy, environmental friendliness, biodegradability, and reduced sludge production.

Chemical coagulants, such as aluminum sulfate, can generate non-biodegradable residues that accumulate in aquatic environments, disrupting ecological cycles, degrading water and soil quality, and posing public health risks. Prolonged exposure to these residues has been linked to neurological and cardiovascular diseases, as highlighted in [4], which addresses the health risks associated with toxic metals in industrial wastewater and emphasizes the role of coagulation-flocculation methods in mitigating these contaminants.

In this context, natural coagulants, especially those from Moringa oleifera, have emerged as a promising solution. Known for its health and nutrition applications, Moringa oleifera has been extensively studied for its coagulant potential. Natural coagulants provide advantages over chemical ones, including environmental biodegradability and non-toxicity in treated water.

Despite these clear benefits, the production of natural coagulants from Moringa oleifera typically involves complex processes, such as microfiltration and lyophilization, as noted in [5], which can be technically challenging and economically unviable in resource-limited areas. The requirement to add saline solutions to extract the active compounds from Moringa oleifera, along with the need for sophisticated equipment, limits the widespread adoption of these methods in vulnerable communities.

Given this, there is a need for methods that not only preserve the efficacy of natural coagulants but also simplify the production process, making it accessible to populations without advanced infrastructure. For example, paper filtration is a low-cost (approximately $0.04 on the U.S. market) and easily implementable technique that can be used to efficiently and rapidly produce natural coagulants. This method eliminates the need for microfiltration and lyophilization, enabling rapid coagulant production in minutes through simple steps: peeling, grinding the Moringa oleifera seeds, stirring with water, and filtering through paper.

Therefore, this paper aims to present an economical, sustainable, and effective method for producing a Moringa oleifera-based natural coagulant using paper filtration. This method enhances water treatment accessibility in vulnerable communities by providing a practical, eco-friendly solution adaptable to various contexts, aiming to improve public health and promote equitable access to quality water while advancing sustainable technologies.

## Method details

This section provides a detailed description of an innovative and simplified method developed for producing a natural coagulant from the seeds of Moringa oleifera using paper filtration. The goal of this method is to offer an economical and sustainable solution for water treatment, particularly in vulnerable communities with limited infrastructure. The process is designed to be accessible, removing the need for complex and expensive techniques that often hinder the application of natural coagulants in resource-constrained areas. The materials and equipment used in the study are described in Table 1, Table 2, respectively, while a detailed description of the proposed methodology is provided in Table 3.

**Table 1:** Materials used in the preparation of the natural coagulant.

Source: Authors (2024).

**Table 2:** Equipment used in the preparation of the natural coagulant.

Source: Authors (2024).

**Table 3:** Steps for preparing the Moringa oleifera-based natural coagulant.

Source: Authors (2024).

## Method validation

To assess the alternative methodology for producing the Moringa oleifera-based natural coagulant, turbidity control was evaluated, as it is a widely used parameter for determining the efficiency of coagulation, flocculation, and sedimentation processes in water treatment systems. All tests were conducted using a jar test, aimed at exploring various operational conditions, such as coagulant type and concentration, across multiple water samples simultaneously. The procedure began with the preparation of synthetic water samples, with turbidity adjusted to predetermined levels (100, 200, and 300 NTU).

**Fig. 1:**
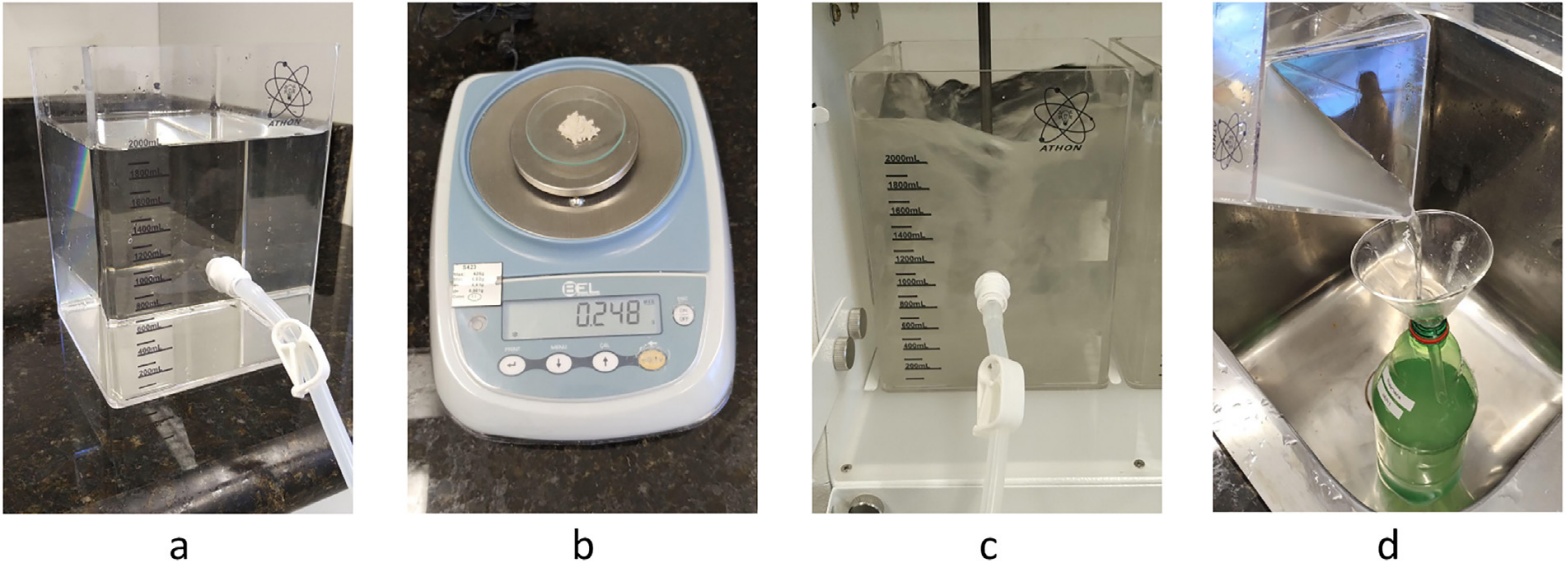
Steps for preparing synthetic water: (a) Tap water was added to the jar test vessel, (b) bentonite was precisely weighed, (c) the mixture was stirred using the jar test apparatus, and (d) the synthetic water was stored.

Source: Authors (2024).

The synthetic water was prepared by adding bentonite to tap water, a process conducted using the jar test apparatus. This procedure was designed to ensure that key characteristics, such as turbidity and pH, remained consistent throughout all tests. Tap water was placed in a 2-liter jar test vessel (Fig. 1a). The bentonite was precisely weighed using a balance, with 0.248 g of bentonite added per 2 liters of water to achieve an average turbidity of 50 NTU (Fig. 1b). The mixture was then stirred in the jar test apparatus for 30 min at 500 rpm to ensure complete dissolution of the bentonite. Following this process, the synthetic water was stored in containers, such as 2-liter bottles, for use in subsequent experiments. After preparation, the synthetic raw water was allowed to settle for at least 24 h, as recommended in [[6], [7]].

Different dosages of the Moringa oleifera-based natural coagulant were then added to each sample, followed by vigorous initial stirring (151 rpm) to ensure uniform distribution of the coagulant and to promote aggregate formation. It is important to highlight that the properties of Moringa oleifera seeds can influence the characteristics of the coagulant and impact the overall efficiency of the process. The potential variability in seed properties, such as those caused by climate or geographic origin, may affect coagulant performance, which could be crucial for ensuring the method's replicability in different regions. After this stage, the samples were allowed to settle, enabling the flocs to sediment and facilitating the removal of impurities from the water. The residual turbidity was subsequently measured, providing a quantitative evaluation of the coagulant's effectiveness under the tested conditions.

Initially, tests were conducted on water samples with an initial turbidity of 200 NTU. Encouraged by the promising results, the analysis was expanded to include turbidity levels of 300 NTU and 100 NTU, with coagulant dosages adjusted in the latter to evaluate efficacy with lower quantities.

## Turbidity Removal in Samples with an Initial Turbidity of 300 NTU

For samples with an initial turbidity of approximately 300 NTU, the initial values and their variations are presented in Table 4. After 10 min of sedimentation, turbidity was reduced to below 22 NTU, indicating a significant reduction of approximately 93 % within the initial minutes. After 20 min, turbidity levels further decreased, ranging from 7.4 NTU to 0.0 NTU, with two jars already exhibiting zero turbidity during this period. By 30 min, most samples achieved zero or near-zero turbidity, with values between 5.2 NTU and 0.0 NTU, remaining stable until the conclusion of the experiment at 60 min.

**Table 4:** Turbidity values for samples with an initial turbidity of 300 NTU, measured over sedimentation times ranging from 0 to 60 min.

Settling time (min)	Turbidity (NTU)					
	Jar #1 (40 mL)	Jar #2 (45 mL)	Jar #3 (50 mL)	Jar #4 (55 mL)	Jar #5 (60 mL)	Jar #6 (65 mL)
0	304.0	301.0	297.0	296.3	306.7	304.7
10	22.0	18.6	11.6	10.8	8.2	7.5
20	7.4	3.6	0.5	0.2	0.0	0.0
30	5.2	2.2	0.0	0.0	0.0	0.0
40	5.8	2.0	0.1	0.0	0.0	0.0
50	6.6	1.9	0.5	0.0	0.0	0.2
60	5.7	1.1	0.0	0.0	0.0	0.0

Source: Authors (2024).

## Turbidity Removal in Samples with an Initial Turbidity of 200 NTU

For samples with an initial turbidity of approximately 200 NTU, a significant reduction in turbidity was observed after 10 min of sedimentation, with values ranging from 14.1 NTU to 9.4 NTU (Table 5). After 20 min, turbidity decreased further, with all samples showing values below 2.1 NTU. At 40 min, there was some variation in turbidity values, but all remained below 3.9 NTU. By the end of the 50-minute sedimentation period, all samples exhibited zero turbidity.

**Table 5:** Turbidity values for samples with an initial turbidity of 200 NTU, measured over sedimentation times ranging from 0 to 60 min.

Settling time (min)	Turbidity (NTU)					
	Jar #1 (40 mL)	Jar #2 (45 mL)	Jar #3 (50 mL)	Jar #4 (55 mL)	Jar #5 (60 mL)	Jar #6 (65 mL)
0	208.3	209.3	211.7	208.3	210.3	206.8
10	14.1	13.0	9.4	10.3	10.4	10.5
20	2.1	1.3	1.0	1.9	0.0	0.7
30	1.4	3.0	1.7	0.6	1.8	3.1
40	2.7	1.7	3.9	0.3	0.8	0.3
50	0.0	0.0	0.0	0.0	0.0	0.0
60	0.0	0.0	0.0	0.0	0.0	0.0

Source: Authors (2024).

## Turbidity Removal in Samples with an Initial Turbidity of 100 NTU

For samples with an initial turbidity of approximately 100 NTU, the initial values are presented in Table 6. After 10 min of sedimentation, turbidity decreased to below 24.9 NTU. By 20 min, turbidity ranged from 10.9 NTU to 1.4 NTU. At 30 min, a more substantial reduction was observed, with most samples displaying near-zero turbidity, which remained consistent until the end of the experiment.

**Table 6:** Turbidity values for samples with an initial turbidity of 100 NTU, measured over sedimentation times ranging from 0 to 60 min.

Settling time (min)	Turbidity (NTU)					
	Jar #1 (10 mL)	Jar #2 (15 mL)	Jar #3 (20 mL)	Jar #4 (25 mL)	Jar #5 (30 mL)	Jar #6 (35 mL)
0	102.2	107.5	105.6	102.5	102.3	102.0
10	24.9	16.4	11.4	9.6	13.9	16.1
20	10.9	3.9	1.7	1.4	2.1	5.0
30	3.9	3.3	1.1	1.8	0.3	1.3
40	3.8	0.1	0.0	0.0	0.0	0.0
50	1.5	0.0	0.0	0.0	0.0	0.0
60	0.0	0.0	0.0	0.0	0.0	0.0

Source: Authors (2024).

## General analysis of results

The results highlight the high efficiency of the natural coagulant in removing turbidity, particularly in samples with higher initial turbidity levels. Across all tested turbidity ranges (100 NTU, 200 NTU, and 300 NTU), significant turbidity reductions were observed within the initial minutes of sedimentation, with values nearing or reaching zero by the end of the 50-minute period. These findings underscore the potential of natural coagulants as viable and effective alternatives to conventional water clarification methods.

This study emphasizes the transformative potential of a simplified method for producing a natural coagulant from Moringa oleifera seeds, particularly for water treatment in resource-limited and vulnerable regions. The research demonstrated that the coagulant produced through this process is highly effective in removing turbidity, achieving 100 % efficiency with a dosage of 5 mL per liter of water. Moreover, it offers a sustainable and cost-effective alternative to traditional chemical coagulants, which are known for their adverse environmental and public health impacts.

A key innovation of this study is the elimination of complex techniques from the coagulant production process, making the technology accessible to communities without advanced infrastructure. The simple procedures involved—such as peeling, grinding, and paper filtration—allow the coagulant to be produced locally in <30 min, making it immediately available for water treatment.

The significance of this work lies in providing a practical and easily implementable solution to a critical global challenge: access to potable water. By combining simplicity, effectiveness, and sustainability, this study contributes to improving living conditions in underserved communities, enhancing public health, and supporting environmental preservation. Furthermore, widespread adoption of this technology could reduce reliance on chemical coagulants, thereby mitigating the risks associated with contamination of aquatic ecosystems and human health. Consequently, this innovative method for producing a natural coagulant from Moringa oleifera represents a major advancement in water treatment, offering a viable and accessible alternative for regions in need.

## Limitations

This study has limitations that should be acknowledged. Firstly, while the proposed method demonstrated scalability, its practical application in larger-scale operations remains to be fully evaluated. Additionally, the long-term storage of the coagulant was not investigated, raising questions about its stability and efficacy over time. The performance of the coagulant in highly polluted or complex water matrices also requires further exploration, as this study primarily focused on turbidity as the sole indicator of process efficiency. Other important parameters were not tested, which may limit the overall assessment of the coagulant's effectiveness (as chemical, bacterial, and heavy metal contaminants). Future research should aim to evaluate additional characteristics of water quality to provide a more comprehensive understanding of the coagulant's performance in diverse conditions.

## CRediT authorship contribution statement

**Danieli Soares de Oliveira:** Conceptualization, Methodology, Validation, Formal analysis, Investigation, Resources, Data curation, Writing – original draft, Writing – review & editing, Visualization, Supervision, Project administration, Funding acquisition. **Raynara Souza do Nascimento:** Investigation, Data curation. **Clainer Bravin Donadel:** Data curation, Writing – original draft, Writing – review & editing.

## Data Availability

Data will be made available on request.
